# Introduction and Rapid Spread of SARS-CoV-2 Omicron Variant and Dynamics of BA.1 and BA.1.1 Sublineages, Finland, December 2021

**DOI:** 10.3201/eid2806.220515

**Published:** 2022-06

**Authors:** Hanna Vauhkonen, Phuoc Truong Nguyen, Ravi Kant, Ilja Plyusnin, Mert Erdin, Satu Kurkela, Hanna Liimatainen, Niina Ikonen, Soile Blomqvist, Kirsi Liitsola, Erika Lindh, Otto Helve, Hanna Jarva, Raisa Loginov, Aino Palva, Tiina Hannunen, Sari Hannula, Mikko Parry, Paula Kauppi, Antti Vaheri, Tarja Sironen, Maija Lappalainen, Carita Savolainen-Kopra, Teemu Smura, Olli Vapalahti

**Affiliations:** University of Helsinki, Helsinki, Finland (H. Vauhkonen, P. Truong Nguyen, R. Kant, I. Plyusnin, M. Erdin, A. Vaheri, T. Sironen, T. Smura, O. Vapalahti);; University of Helsinki and Helsinki University Hospital, Uusimaa, Finland (S. Kurkela, H. Liimatainen, R. Loginov, M. Parry, P. Kauppi, M. Lappalainen, T. Smura, O. Vapalahti);; Finnish Institute for Health and Welfare (THL), Helsinki (N. Ikonen, S. Blomqvist, K. Liitsola, E. Lindh, O. Helve, C. Savolainen-Kopra);; Institute for Molecular Medicine Finland (FIMM), Helsinki (A. Palva, T. Hannunen, S. Hannula)

**Keywords:** COVID-19, SARS-CoV-2, SARS-CoV-2 Omicron variant, SARS-CoV-2 Omicron sublineages BA.1 and BA.1.1, disease spread, Finland, viruses, zoonoses, respiratory infections

## Abstract

Multiple introductions of SARS-COV-2 Omicron variant BA.1 and BA.1.1. lineages to Finland were detected in early December 2021. Within 3 weeks, Omicron overtook Delta as the most common variant in the capital region. Sequence analysis demonstrated the emergence and spread through community transmission of a large cluster of BA.1.1 virus.

The most recent SARS-CoV-2 variant of concern, Omicron (Pango lineage B.1.1.529), was first detected in South Africa (*1*), although it might have emerged elsewhere, and has since spread globally at an unforeseen speed. Notable examples include a superspreading event in Norway (*2*) and the rapid increase in incidence in Denmark (*3*) despite high vaccination coverage (83% of infected persons had received 2–3 vaccine doses). This rapid spread indicates the novel variant’s exceptional transmissibility, as well as its potential for reinfection and vaccination breakthrough. We describe the genotypes of cases of Omicron entering Finland from their early spread up to established community transmission through the first week of January 2022. No ethics approval was needed because this study was based on routine COVID-19 surveillance data. The study regarding Helsinki University Hospital (HUH) samples was approved by the local ethical and research committee (Helsinki and Uusimaa Hospital District [HUS]; Clinical microbiology of COVID-19: diagnostics, laboratory findings and biorisks; HUS/244/2021). 

## The Study

**Figure 1:**
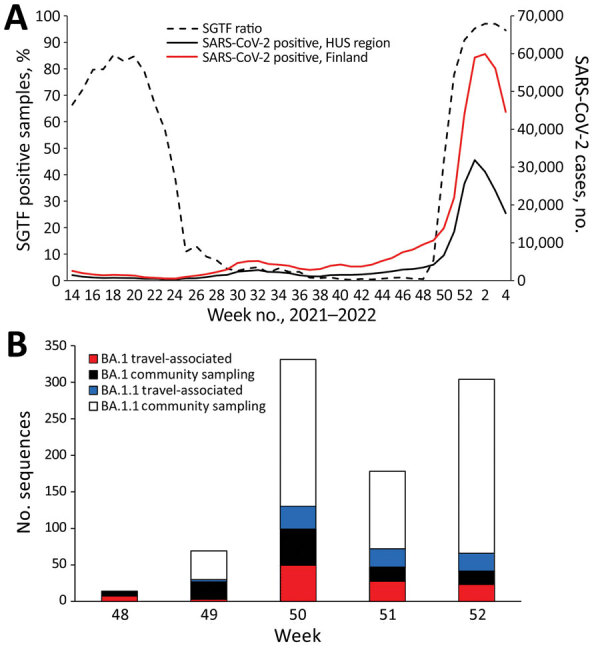
Introduction and spread of SARS-CoV-2 Omicron variant in Finland in late 2021–early 2022. A) Confirmed SARS-CoV-2 positives in Finland (red) and in the HUS region (black) and the proportion of SGTF measured by reverse transcription PCR–positive cases analyzed by the HUS Clinical Microbiology division (dashed line) from week 14 in 2021 through week 4 in 2022 (National Infectious Disease Registry, https://www.thl.fi/ttr/gen/rpt/tilastot.html). B) Weekly numbers of travel-associated and community sampling–derived Omicron cases (Pango lineages BA.1 and BA.1.1) for weeks 48–52, 2021. Travel-associated status was defined by either being sampled at a border or a patient record indicating most likely country of infection abroad. The lower amount of sequences obtained for week 51 originates most likely from the Christmas holiday season. Week 52 was the last full week of our study period. HUS, Helsinki and Uusimaa Hospital District; SGTF, S-gene target failure

A total of 99,988 samples found positive for SARS-CoV-2 by reverse transcription PCR, 12.1% of 825,006 total samples tested, were detected in Finland during the study period, November 29, 2021–January 6, 2022 (Figure 1). Weekly positivity rates among persons tested rose from 6.1% of 156,077 in week 48 to 25.6% of 172,451 (3.1% of the Finnish population) in week 52 (https://sampo.thl.fi). In HUS, test positivity increased from 5.0% to 36.7% over the corresponding weeks 48–52. After a change in testing strategy favoring home antigen testing, the number of registered SARS-CoV-2 cases dropped (Appendix). 

We estimated the proportions of Omicron variant lineages BA.1 and BA.1.1 within HUS by comparing PCR-based data on S-gene target failure (SGTF) to that of other circulating lineages (Figure 1; Appendix). The results showed a decrease in SGTF rates from week 24, when the proportion of the Alpha variant (B.1.1.7) was declining, to near 0 when the Delta variant (B.1.617.2) was dominant. This decrease aligns well with sequence-confirmed lineage turnover reported elsewhere (*4*). Thereafter, the proportion of SGTF rose steeply from week 48 of 2021 until it reached 97% in week 2 of 2022 (Figure 1), indicating a rapid spread of the BA.1 and BA.1.1 lineages in the capital region of Finland.

The sequenced samples consisted of randomly selected population samples and samples collected at border entry (through airports, harbors, and land borders) to Finland (Table). In addition, a small proportion was preselected based on SGTF positivity (Appendix). Omicron sequence data consisted of 962 sequences, 33.4% of all sequenced samples (n = 3,100; ≈2% of all confirmed cases), during November 29, 2021–January 6, 2022. We collected 133 samples at points of border entry and recorded the number of patients in each hospital district, demographic distribution, and travel status (Table), including countries of origin for the travel-associated cases (Appendix). In addition, we added 15 Omicron sequences obtained from hospitalized patients in HUH to the sequence dataset (Appendix). 

**Table:** Patient data for 979 sequenced Omicron genomes in investigation of SARS-CoV-2 Omicron variant and dynamics of BA.1 and BA.1.1 sublineages, Finland, December 2021*

Variables		No. (%)†
Sex		
M		513 (52.4)
F		466 (47.6)
Travel		
Abroad		57 (5.8)
Border‡		20 (2.0)
Finland		234 (23.9)
Border‡		6 (0.6)
NA§		688 (70.3)
Border‡		107 (10.9)
Age, y		
Range		0–98
Mean		36.2
Median		34
Sample origin		
HUS		662 (79.7)
Non-HUS total¶		169 (20.3)
Non-HUS by district, no.		
Central Finland Health Care District		6
Central Ostrobothnia Hospital District		6
East Savo Hospital District		3
Hospital District of South Ostrobothnia		10
Hospital District of Southwest Finland		18
Kainuu Social and Health Care Joint Authority		5
Tavastia Proper Hospital District		3
Kymenlaakso Social and Health Services		7
Lapland Hospital District		4
North Karelia Social and Health Care Authority		17
North Ostrobothnia Hospital District		15
North Savo Hospital District		12
Pirkanmaa Hospital District		10
Päijät-Häme Hospital District		3
Satakunta Hospital District		20
South Karelia Social and Health Care District		12
South Savo Social and Health Care Authority		5
Vaasa Hospital District		6
Åland Hospital District		7
Other sample origin		
HUH		15 (1.5)
Border		133 (13.6)

*NA, not available; HUH, Helsinki University Hospital; HUS, Helsinki and Uusimaa Hospital District; Non-HUS, hospital district other than HUS. †Unless otherwise indicated. ‡Border, samples collected from border entry (airports, harbors, and land). §Travel data not available, probably originating from Finland. ¶Other, sample collection based on other than hospital districts.

We identified Omicron cases in 5 travelers returning to Finland from Sweden through Denmark during November 29–30, 2021. All 5 members of the travel party, who lived in 3 different hospital districts (HUS, Hospital District of Southwest Finland, and North Savo Hospital District), were found to be Omicron positive through PCR testing and sequencing. The identified sequences clustered together with reference sequences mainly from Denmark and Sweden. However, introduction from this travel party did not lead to wide community circulation.

After the first introduction events, the number of weekly sequence-confirmed Omicron cases rose sharply during weeks 49 and 50 (Figure 1, panel B). Although weekly numbers of travel-associated (most likely imported) cases of lineage BA.1 did not differ from those for BA.1.1 (χ^2^ = 1.03; p = 0.5975), the proportion of BA.1.1 in the community samples was significantly higher than that of BA.1 (2-sample z-test, p = 0.0024, week 49 vs. week 50; (Appendix). We did not detect lineage BA.2. during the study period.

**Figure 2:**
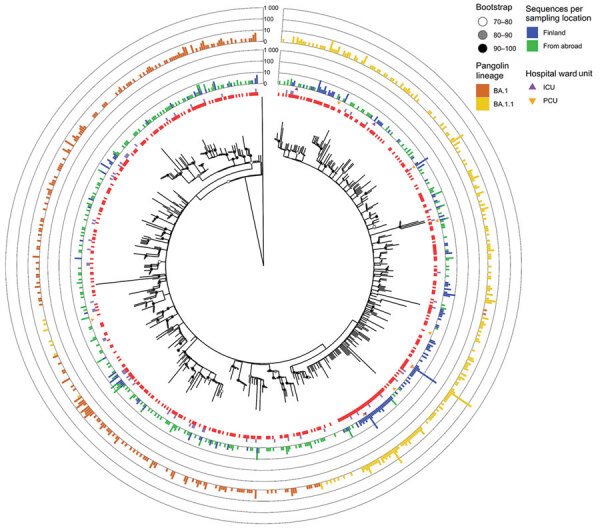
Clustering analysis of Omicron sequences in study of SARS-CoV-2 Omicron variant in Finland in late 2021–early 2022. The collapsed maximum-likelihood phylogenetic tree shows Omicron genomes sampled in Finland (n = 870) and reference sequences from other countries (n = 754), the reference dataset we used. The outermost bar plot shows the number of BA.1 and BA.1.1 sequences in each cluster. Purple squares indicate Omicron sequences collected from a Finland border; clusters with border samples each contain 1–9 sequences. Clustering analysis revealed that, by the beginning of January 2022, aside from 1 major BA.1.1 cluster (n = 236, 27.1% of all cases in Finland during the study period, November 29, 2021–January 6, 2022), most (n = 634, 72.8% of cases) Omicron cases in Finland were either singletons or small clusters (≤30 sequences). The tree was inferred using the IQTREE2 version 2.0.6 (http://www.iqtree.org) using ModelFinder and 1,000 bootstraps were computed with the integrated Ultrafast bootstrap algorithm and the clusters (red squares) with TreeCluster version 1.0.3 (https://github.com/niemasd/TreeCluster) using an arbitrary branch length of 0.001 and support value of 70. Triangles indicate sequences recorded from patients in the ICU or PCU. The tree is rooted to an Omicron BA.2 sequence (Genbank accession no. OV698431.1). ICU, intensive care unit; PCU, pulmonary care unit

Our phylogenetic and clustering analysis (Figure 2) inferred 80 small, highly supported lineage BA.1 subclusters that contained sequences (n = 168) from Finland, as well as 47 BA.1 sequences that were singletons or from low-support clusters. For BA.1.1 sequences, the analysis inferred 129 clusters containing BA.1.1 sequences (n = 570) from Finland and 75 singletons. Of note, among BA.1.1 clusters, 1 cluster contained 236 identical sequences, 24.5% of all Omicron sequences from Finland recorded during the study period. These sequences were also identical to isolate HKU-344 (OM212473) from Hong Kong, collected November 27, 2021. These identical sequences were detected starting December 7 through the end of the study period. Most of these cases, 197/236 (83.5%), were detected in HUS, including the first 2 cases on December 7. Eleven of the sequences from this clade were imported, with the most likely countries of infection reported as Estonia (December 9, 2021), Sweden (December 15), and the United Kingdom, Spain, or Portugal (all December 20). An additional 8 cases were sampled at the border during December 15–21; 1 originated from Sweden, but no data were available about the country of infection for the other cases. Although the analysis of imported cases suggested that a virus of identical genotype was circulating in several European countries, locally acquired infections of this genotype were detected before the documented importation events.

Altogether the results suggest widescale rapid spread of BA.1.1 in Finland. COVID-19 patients hospitalized at HUH pulmonary or intensive care units showed similar, albeit delayed, lineage turnover from Delta variant to Omicron variant (Appendix), consistent with population-level data. 

## Conclusions

We characterize the rapid increase in incidence of the SARS-CoV-2 Omicron variant in Finland. Specifically, our data suggest that BA.1.1 rapidly emerged as the dominant lineage over its parent, BA.1. The BA.1.1 lineage-defining R346K substitution in the spike protein has been suspected of increasing transmission rates more than the BA.1 lineage. This substitution, which occurred convergently in the Mu variant of concern, provides evidence of positive selection (*1*,*5*) and affects antibody binding (*6*). Although this mutation might provide an additional transmission advantage through enhanced immune-escape properties in a population, alternative options such as the founder effect cannot be ruled out for explaining the rapidly established dominance of this lineage in Finland. 

Overall, Finland represents one of the countries with a rapid surge of Omicron variant BA.1.1 lineage introduced into a population largely vaccinated with 2 shots and within an epidemiologic landscape of increasing Delta circulation and absent or very low BA.2 circulation. These dynamics resulted in the dominance of BA.1.1 over both the Omicron BA.1 and Delta strains. Our study exemplifies the need for genomic surveillance and rapid detection of emerging SARS-CoV-2 lineages to support public health response and mitigation efforts.

Appendix Additional information on the introduction and spread of SARS-CoV-2 Omicron BA.1 and BA.1.1 variants in Finland
