# Supplementary material for: Introduction and Rapid Spread of SARS-CoV-2 Omicron Variant and Dynamics of BA.1 and BA.1.1 Sublineages, Finland, December 2021
Source: Emerg Infect Dis. 2022 Jun;28(6):1229–32. doi: 10.3201/eid2806.220515 (PMC9155872; doi:10.3201/eid2806.220515)
Supplement: Appendix — Additional information on the introduction and spread of SARS-CoV-2 Omicron BA.1 and BA.1.1 variants in Finland [file 22-0515-Techapp-s1.pdf]

Article DOI: https://doi.org/10.3201/eid2806.220515

Introduction and Rapid Spread of SARS-CoV-2 Omicron Variant and Dynamics of BA.1 and BA.1.1 Sublineages, Finland, December 2021

Appendix

SARS-CoV-2 testing

During the study period, 29 Nov 2021 to 6 Jan 2022, SARS-CoV-2 was tested by reverse transcriptase PCR (RT-PCR) on three different occasions: 1) from travelers entering the country (https://raja.fi/en/guidelines-for-border-traffic-during-pandemic), 2) by testing asymptomatic people needing travel documentation and 3) symptom-based testing according to recommendations of each hospital district in Finland. Until the explosive rise in SARS-CoV-2 Omicron infections, the testing capacity was able to handle the symptom-based testing and contact tracing was partially able to resolve chains of transmission. However, with case numbers rising exponentially towards the end of 2021, healthy individuals with mild COVID-19 symptoms were recommended to perform lateral flow antigen testing on their own and stay home. Therefore, the reported COVID-19 cases might be underreported.

SARS-CoV-2 testing by RT-PCR was conducted in diagnostic laboratories throughout Finland. For S-gene target failure (SGTF, a dropout of the S-gene PCR product due to deletion targeting residues 69-70 in the spike protein coding region), Thermo Fisher TaqPath COVID-19 assay was used to analyze samples received by HUS Clinical Microbiology division. The assay was able to S-gene target failure is typical for Omicron variant lineages BA.1/BA.1.1. Since other SARS-CoV-2 lineages with deletion in this genome region (such as Alpha variant) have not been detected in Finland since early autumn of 2021, a sample with SGTF was considered as an Omicron BA.1/BA.1.1 suspect.

SARS-Cov-2 sequencing

Samples subjected to sequencing were collected by HUS Clinical Microbiology division and Finnish Institute for Health and Welfare (THL), and were sent to University of Helsinki for sequencing. The samples originated throughout Finland. In HUS Clinical Microbiology division, a subset of Omicron-suspected SGTF samples were selected for sequencing, as well as a all positive border entry samples and random subset of community samples (the latter two irrespective of SGTF status, i.e. not biased towards Omicron positivity). The sequenced subset of positive cases collected by THL was based on random community samples with a few exceptions of known contacts of the first Omicron positive cases detected in Finland. The RT-PCR of these samples was carried out in local diagnostic laboratories and selection for sequencing was done irrespective of SGTF status.

The Helsinki University Hospital (HUH) data set (Appendix Table) was obtained from patients who were receiving care on 7 January 2022 for a PCR-diagnosed COVID-19 infection in either Pulmonary Diseases ward or ICU (Appendix Table), resulting in altogether 15 sequenced Omicron and 15 Delta genomes. Based on SGTF status of the initial RT-PCR testing, the final Omicron and Delta sample numbers were 19 and 18, respectively. All patients were alive on data collection day Jan 25 2022 and were of Finnish origin with no travel abroad, except one resident of Poland with no available travel data.

RNA was extracted from nasopharyngeal swab specimens using either the MagNA Pure 96 Instrument (RocheMolecular Systems Inc. Plesanton, CA, USA) or QIAamp Viral RNA Kit (Qiagen, Hilden, Germany). cDNA synthesis was conducted using Lunascript RT SuperMix Kit (New England Biolabs, Ipswich, MA) with random hexamers and oligo dT. SARS-CoV-2-specific amplicons were generated using xGen Artic V4 NCoV-2019 primers (Integrated DNA Technologies Inc., Coralville, Iowa) spiked with additional primers designed for Omicron amplification (https://community.artic.network/t/sars-cov-2-v4-1-update-for-omicron-variant/342) using Q5 Hot Start HighFidelity 2X Master Mix (New England Biolabs). The PCR amplicons were purified using Optima DTR 96-well clean-up system (Edge BioSystems, San Jose, CA, USA), end-prepped with NEBNext End Prep enzyme (New England Biolabs), ligated with unique dual indexes (Integrated DNA Technologies Inc.) using NEBNext Ultra II Ligation Module (New England Biolabs), pooled with 48 or 96 samples in one pool and purified using SpriSelect beads (Beckman Coulter Life Sciences, Indianapolis, IN, USA). The index-ligated

amplicons were amplified with KAPA HiFi HotStart ReadyMix (Roche Sequencing Solutions Inc, Pleasanton, CA, USA) and standard Illumina P5 and P7 primers (P5, AATGATACGGCGACCACCGAGATCT and P7, CAAGCAGAAGACGGCATACGAGAT) and purified with SpriSelect beads (Beckman Coulter Life Sciences). The pools were quantitated using Qubit fluorometer (Thermo Fisher Scientific, Waltham, MA, USA) and sequenced either with Illumina MiSeq system using the v3 sequencing kit (600 cycles) or Illumina NovaSeq 6000 system with NovaSeq 6000 SP Reagent Kit v1.5 (500 cycles).

Sequence analysis

The metadata on sequence-confirmed Omicron cases was retrieved from the Finnish National Infectious Disease Register on Jan 21 2022. The data consisted of 964 cases collected between Nov 29 2021 and Jan 6 2022. Of these, 133 samples were collected at points of border entry (airports, harbors and land borders). Numbers of patients in each hospital district, demographic distribution and travel records are shown in Table 1.

The raw sequence reads were trimmed, quality filtered and assembled using fastp (*1*) and BWA-MEM (*2*) programs implemented in HAVoC pipeline (*3*). The lineage assignment was conducted using the pangolin tool (v 3.1.20) (*4*).

The sequences with less than 1 700 ambiguous nucleotide positions were included in the phylogenetic analysis, resulting in 870 Omicron sequences from Finland. A global subsample of Omicron sequences was constructed by identifying sequences closely related to the Finnish Omicron sequences using UShER tool (*5*) and retrieving these from GenBank and Gisaid databases. The global sequence data was further downsampled by removing identical sequences and accepting one representative of each clade per country.

The omicron sequences were aligned with nextalign (v 1.11.0) package from Nextclade (*6*) and the phylogenetic trees were inferred with maximum likelihood method implemented in IQTREE2 (v. 2.0.6) software (*7*) using ModelFinder (*8*) and 1 000 bootstrap replicates were computed with Ultrafast bootstrap algorithm (*9*). Cluster assignment was conducted with TreeCluster (v 1.0.3) (*10*) using an arbitrary branch length of 0.001 and support value of 70.

The statistical analyses were carried out using the web-based software Epitools (https://epitools.ausvet.com.au).

References

1. Chen S, Zhou Y, Chen Y, Gu J. fastp: an ultra-fast all-in-one FASTQ preprocessor. Bioinformatics. 2018;34:i884–90. PubMed https://doi.org/10.1093/bioinformatics/bty560

2. Li H. Aligning sequence reads, clone sequences and assembly contigs with BWA-MEM. arXiv. 2013.

3. Truong Nguyen PT, Plyusnin I, Sironen T, Vapalahti O, Kant R, Smura T. HAVoC, a bioinformatic pipeline for reference-based consensus assembly and lineage assignment for SARS-CoV-2 sequences. BMC Bioinformatics. 2021;22:373. PubMed https://doi.org/10.1186/s12859-021-04294-2

4. O'Toole Á, Scher E, Underwood A, Jackson B, Hill V, McCrone JT, et al. Assignment of epidemiological lineages in an emerging pandemic using the Pangolin tool, Virus Evolution 2021;7,veab064.

5. Turakhia Y, Thornlow B, Hinrichs AS, De Maio N, Gozashti L, Lanfear R, et al. Ultrafast Sample placement on Existing tRees (UShER) enables real-time phylogenetics for the SARS-CoV-2 pandemic. Nat Genet. 2021;53:809–16. PubMed https://doi.org/10.1038/s41588-021-00862-7

6. Aksamentov I, Cornelius Roemer C, Hodcroft EB. 3, Neher RA. Nextclade: clade assignment, mutation calling and quality control for viral genomes. J Open Source Softw. 2021;6:3773. https://doi.org/10.21105/joss.03773

7. Minh BQ, Schmidt HA, Chernomor O, Schrempf D, Woodhams MD, von Haeseler A, et al. IQ-TREE 2: new models and efficient methods for phylogenetic inference in the genomic era. Mol Biol Evol. 2020;37:1530–4. PubMed https://doi.org/10.1093/molbev/msaa015

8. Kalyaanamoorthy S, Minh BQ, Wong TKF, von Haeseler A, Jermiin LS. ModelFinder: fast model selection for accurate phylogenetic estimates. Nat Methods. 2017;14:587–9. PubMed https://doi.org/10.1038/nmeth.4285

9. Hoang DT, Chernomor O, von Haeseler A, Minh BQ, Vinh LS. UFBoot2: Improving the ultrafast bootstrap approximation. Mol Biol Evol. 2018;35:518–22. PubMed https://doi.org/10.1093/molbev/msx281

10. Balaban M, Moshiri N, Mai U, Jia X, Mirarab S. TreeCluster: clustering biological sequences using phylogenetic trees. PLoS One. 2019;14:e0221068. PubMed https://doi.org/10.1371/journal.pone.0221068

Appendix Table. Helsinki University Hospital SARS-CoV-2 positive patients receiving hospital care on January 7, 2022 on either pulmonary care unit or intensive care unit hospital ward*

Patient	Sequence†	SGTF‡	Sampling date	Age	Sex	Ward§	Ward stay#, d
1	Delta	non-SGTF	2021 Dec 2	56	F	PCU	> 48/22
2	Delta	non-SGTF	2021 Dec 17	59	F	PCU	13/5
3	Delta	non-SGTF	2021 Dec 18	45	M	PCU	22/15
4	Omicron	SGTF	2021 Dec 21	73	M	PCU	21/0
5	NA	non-SGTF	2021 Dec 22	70	F	PCU	12/0
6	Delta	NA	2021 Dec 24	49	M	PCU	10/5
7	Delta	NA	2021 Dec 28	45	F	PCU	11/9
8	NA	SGTF	2021 Dec 30	29	F	PCU	5/0
9	NA	SGTF	2021 Dec 30	72	F	PCU	8/0
10	NA	SGTF	2021 Dec 31	56	M	PCU	8/0
11	NA	non-SGTF	2021 Dec 31	45	F	PCU	8/5
12	Omicron	NA	2022 Jan 3	77	F	PCU	18/0
13	Omicron	NA	2022 Jan 4	72	F	PCU	4/0
14	Delta	NA	2022 Jan 5	63	M	PCU	7/0
15	Omicron	NA	2022 Jan 6	66	M	PCU	>20/11
16	Delta	NA	2022 Jan 6	42	M	PCU	9/0
17	NA	SGTF	2022 Jan 1	68	F	PCU	3/0
18	Omicron	NA	2022 Jan 6	59	M	PCU	4/0
19	Omicron	NA	2022 Jan 6	83	F	PCU	7/0
20	Omicron	NA	2021 Dec 26	70	F	PCU	17/0
21	Omicron	NA	2021 Dec 30	70	M	PCU	9/0
22	Omicron	NA	2022 Jan 6	63	M	PCU	>20/4
23	Omicron	NA	2021 Dec 26	67	M	PCU	13/0
24	Omicron	NA	2022 Jan 4	61	F	PCU	7/0
25	Delta	NA	2022 Jan 6	42	F	PCU	7/0
26	Delta	NA	2022 Jan 2	35	M	PCU	10/0
27	Delta	non-SGTF	2021 Dec 3	56	F	ICU	>49/>48
28	Delta	non-SGTF	2021 Dec 14	66	M	ICU	28/13
29	Delta	non-SGTF	2021 Dec 21	59	M	ICU	19/11
30	Omicron	NA	2021 Dec 29	61	F	ICU	13/6
31	Delta	NA	2022 Jan 6	72	M	ICU	>21/>20
32	NA	non-SGTF	2022 Jan 1	47	M	ICU	11/7
33	Omicron	NA	2022 Jan 2	73	M	ICU	14/7
34	Omicron	NA	2022 Jan 6	60	M	ICU	>20/>20
35¶	Delta	NA	2021 Dec 20	35	M	ICU	>37/>35
36	Delta	NA	2021 Dec 31	70	F	ICU	>26/>25
37	Omicron	NA	2022 Jan 4	48	M	ICU	22/10

*SGTF, S-gene target failure; ICU, Intensive care unit, PCU, pulmonary care unit; NA, not available; SARS-CoV-2, severe acute respiratory syndrome coronavirus 2; d, days. †Sequence, SARS-CoV-2 sequence, Delta, Pangolin lineage B.1.617.2, Omicron, Pangolin lineage B.1.1.529. ‡SGTF in the initial SARS-Cov-2 RT-PCR. §Ward, Hospital ward unit on Jan 7 2022. ¶Patient with non-Finnish origin (Poland), travel status nor known. #Ward stay, length of stay at hospital ward/ICU ward.

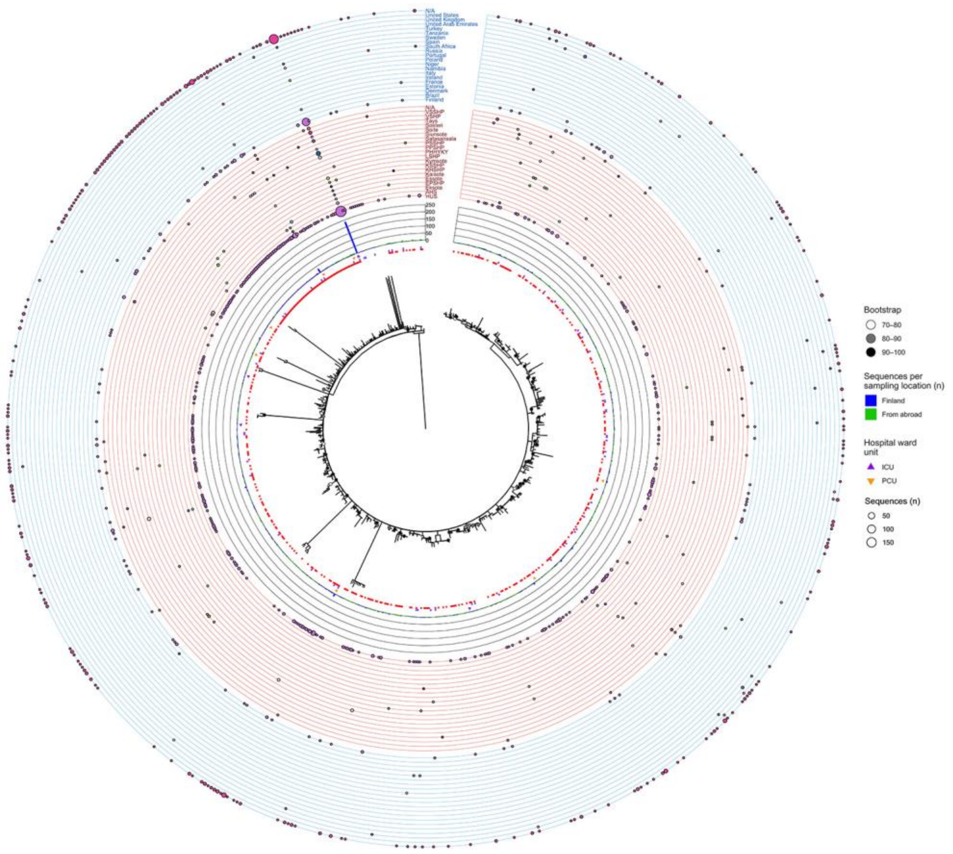

Appendix Figure 1. Clustering analysis of Finnish Omicron sequences. A collapsed maximum-likelihood phylogenetic tree of Omicron genomes sampled in Finland (n = 870) and reference sequences from abroad (n = 754), i.e. reference dataset. These are separated in the barplot in blue and green, respectively. The number of BA.1 and BA.1.1 sequences in each cluster are shown in the outermost barplot. Omicron sequences collected from the Finnish border are shown with purple squares. Clusters with border samples contain at most 1–9 sequences each. Clustering analysis reveals that by the beginning of January 2022, aside from one major BA.1.1 cluster (n = 236, which is 27.1% of all Finnish cases), the large majority of Omicron cases in Finland (n = 634, 72.8% of cases) were either singletons or minute clusters (≤30 sequences). The sampling location for each Omicron case in each cluster is shown with circles in two grids. The size of the circles indicate the number of sequences (n) from Finnish hospital districts (red grid) and from countries of infection (blue grid). Most of the sequences constituting the major clusters originated from local infections in Finland and were sampled in the Hospital District of Helsinki and Uusimaa (HUS). The tree was inferred with the IQTREE2 (v. 2.0.6) using ModelFinder and 1 000 bootstraps were computed with the integrated Ultrafast bootstrap algorithm, and the clusters (red squares) with TreeCluster (v. 1.0.3) using an arbitrary branch length of 0.001 and support value of 70. Sequences recorded from patients that were either in the intensive care unit (ICU) or pulmonary care unit (PCU) are indicated with triangles. The tree is rooted to a Omicron BA.2 sequence (Genbank: OV698431.1). Hospital district glossary: ÅHS = Åland Hospital District, Eksote = South Karelia Social and Health Care District, EPSHP = Hospital District of South Ostrobothnia, Essote = South Savo Social and Health Care Authority,

KHSHP = Tavastia Proper Hospital District, KSSHP = Central Finland Health Care District, Kymsote = Kymenlaakso social and health services, LSHP = Lapland Hospital District, PHHYKY = Päijät-Häme Hospital District, PPSHP = North Ostrobothnia Hospital District, PSSHP = North Savo Hospital District, Soite = Central Ostrobothnia Hospital District, Tays = Pirkanmaa Hospital District, VSHP = Vaasa Hospital District, Sosteri = East Savo Hospital District, Kaisote = Kainuu Social and Health Care Joint Authority, Siunsote = North Karelia Social and Health Care Authority, Satasairaala = Satakunta Hospital District, VSSHP = Southwest Finland Hospital District.

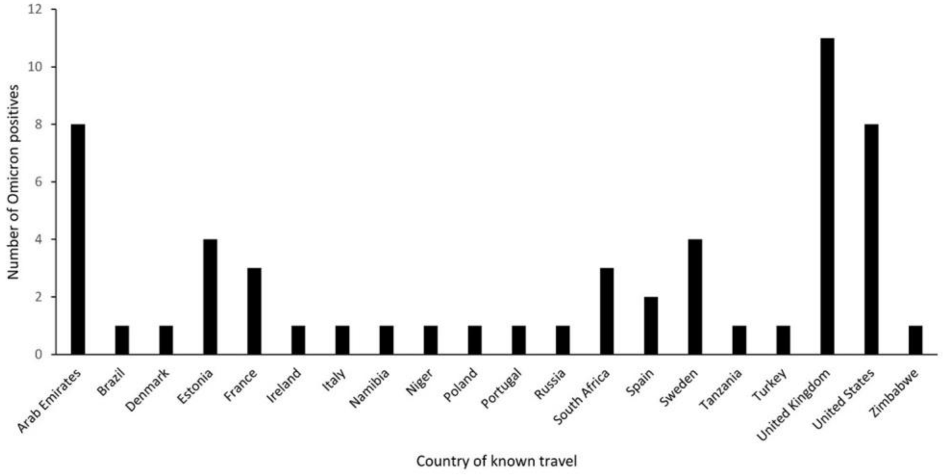

Appendix Figure 2. Finnish Omicron sequences with known traveling abroad. Travel data was obtained from 291/964 cases, of which 57 were reported abroad. 234 cases reported only domestic travel.

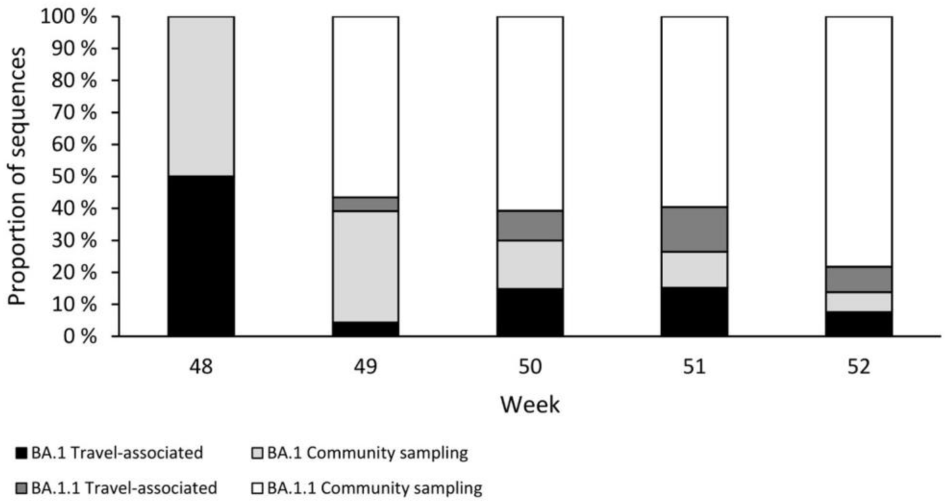

Appendix Figure 3. Weekly proportions of travel-associated and community sampling-derived Omicron variant lineages BA.1 and BA.1.1. Travel-associated was defined as sampled either at border or patient record indicating most likely country of infection abroad.

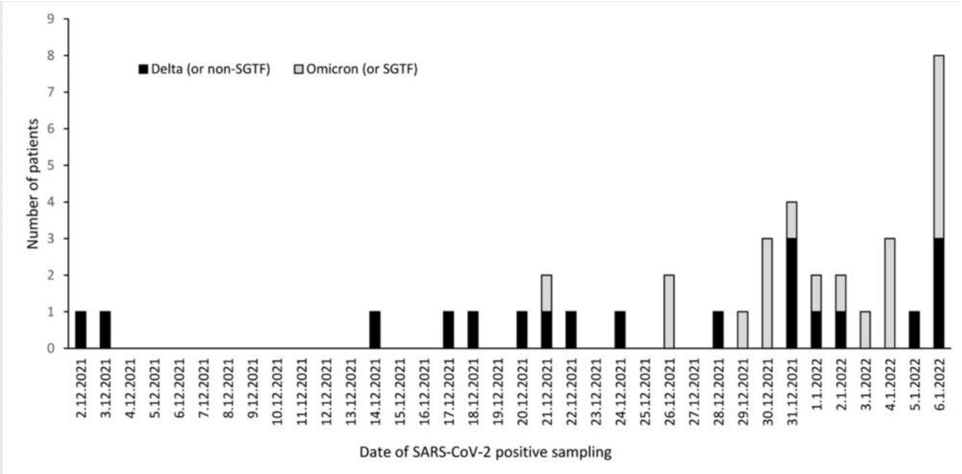

Appendix Figure 4. The onset of disease of the patients in the Pulmonary Care Unit or Intensive Care Unit with Omicron and Delta variants.
